# Poor response to methylphenidate is associated with a smaller dorsal attentive network in adult Attention-Deficit/Hyperactivity Disorder (ADHD)

**DOI:** 10.1038/s41398-023-02598-w

**Published:** 2023-09-30

**Authors:** Valeria Parlatini, Joaquim Radua, Aleix Solanes Font, Rob Wichers, Stefanos Maltezos, Masafumi Sanefuji, Flavio Dell’Acqua, Marco Catani, Michel Thiebaut de Schotten, Declan Murphy

**Affiliations:** 1 Sackler Institute of Translational Neurodevelopment, Department of Forensic and Neurodevelopmental Sciences, Institute of Psychiatry, Psychology and Neuroscience, King’s College London, SE5 8AF London, UK; 2 Department of Forensic and Neurodevelopmental Sciences, Institute of Psychiatry, Psychology and Neuroscience, King’s College London, SE5 8AF London, UK; 3 Institut d’Investigacions Biomediques August Pi i Sunyer, CIBERSAM, Instituto de Salud Carlos III, University of Barcelona, Barcelona, Spain; 4 Research Centre for Environment and Developmental Medical Sciences, Kyushu University, Fukuoka, Japan; 5 Department of Neuroimaging, Institute of Psychiatry, Psychology and Neuroscience, King’s College London, SE5 8AF London, UK; 6 Biomedical Research Centre for Mental Health at South London and Maudsley NHS Foundation Trust and King’s College London, Institute of Psychiatry, Psychology and Neuroscience, King’s College London, SE5 8AF London, UK; 7 Brain Connectivity and Behaviour Group, Sorbonne Universities, Paris, France; 8 Groupe d’Imagerie Neurofonctionnelle, Institut des Maladies Neurodégénératives-UMR 5293, CNRS, CEA University of Bordeaux, Bordeaux, France

**Keywords:** ADHD, Neuroscience, Predictive markers

## Abstract

Stimulants, such as methylphenidate (MPH), are effective in treating attention-deficit/hyperactivity disorder (ADHD), but there is individual variability in response, especially in adults. To improve outcomes, we need to understand the factors associated with adult treatment response. This longitudinal study investigated whether pre-treatment anatomy of the fronto-striatal and fronto-parietal attentional networks was associated with MPH treatment response. 60 adults with ADHD underwent diffusion brain imaging before starting MPH treatment, and response was measured at two months. We tested the association between brain anatomy and treatment response by using regression-based approaches; and compared the identified anatomical characteristics with those of 20 matched neurotypical controls in secondary analyses. Finally, we explored whether combining anatomical with clinical and neuropsychological data through machine learning provided a more comprehensive profile of factors associated with treatment response. At a group level, a smaller left dorsal superior longitudinal fasciculus (SLF I), a tract responsible for the voluntary control of attention, was associated with a significantly lower probability of being responders to two-month MPH-treatment. The association between the volume of the left SLF I and treatment response was driven by improvement on both inattentive and hyperactive/impulsive symptoms. Only non-responders significantly differed from controls in this tract metric. Finally, our machine learning approach identified clinico-neuropsychological factors associated with treatment response, such as higher cognitive performance and symptom severity at baseline. These novel findings add to our understanding of the pathophysiological mechanisms underlying response to MPH, pointing to the dorsal attentive network as playing a key role.

## Introduction

Attention-deficit/hyperactivity disorder (ADHD) is a neurodevelopmental condition characterized by inattentive and/or hyperactive-impulsive symptoms, which is commonly diagnosed in childhood but persists in adulthood in 40–50% of cases [1]. It therefore remains relatively common in adulthood—with the population prevalence in adults being ~4% [2]. Stimulants, such as methylphenidate (MPH), represent the first line treatment [3] and act by modulating dopamine and norepinephrine transmission in striato-cortical regions [4]. This in turn optimizes regional brain activation and connectivity supporting cognitive functions such as attention and inhibition [5, 6]. MPH has been proven to be effective in reducing ADHD core symptoms, but randomized controlled trials have reported that more than one-third of adults with ADHD fail to respond [7]. Recent meta-analytic evidence also confirmed lower response rates in adults than in children [8], which may be linked to an age-dependent decline in brain plasticity [9, 10]. This is of concern, as untreated ADHD in adults increases the risks of academic and occupational failure, substance abuse, and criminal behavior [11, 12]. Yet, despite these possible outcomes, relatively little is known about the biological mechanisms underlying variation in adult treatment response. Further, our understanding of the neurobiology of ADHD is not yet sufficient to guide prescribing and, in clinical practice, the selection of the most appropriate medication is made on a trial-and-error basis. This means that individuals often need to undergo multiple treatments before achieving a clinical response, an approach that delays recovery; exposes people to an increased risk of side effects; and ultimately is not cost-effective. There is therefore a pressing need to better understand what the factors associated with treatment response are; and where possible at an individual level [13]. This may be particularly relevant to ADHD, as it is a neurodevelopmental condition characterized by substantial clinical and biological heterogeneity and, often, by life-long impairment.

Factors associated with treatment response in ADHD potentially include clinical, neuropsychological, and neurobiological characteristics [14, 15]. Clinical studies have reported that MPH dose, treatment adherence, and psychiatric comorbidity influence treatment outcomes in ADHD; whereas gender and age do not [14]. Some studies have suggested that a higher intelligence quotient (IQ) may be associated with better outcomes, but this was not confirmed by other reports [14, 16]. Nonetheless, some prior studies reported that better performance in specific cognitive domains (e.g. sustained attention as measured by the continuous performance task (CPT)), was associated with improved treatment response [16, 17]. Research into neurobiological factors associated with treatment response has produced inconsistent findings. For instance, some genetic studies reported an association between variants of the dopamine transporter gene (DAT1) and treatment response, but others did not [18]. Nuclear medicine approaches suggested that components of the dopaminergic system, such as DAT availability, may be related to treatment response in adults with ADHD, but this needs further replication in larger samples and may have limited applicability in the ‘real world’ due to the use of ionizing radiation [19, 20]. Magnetic resonance imaging (MRI) studies in children with ADHD that compared ‘responders’ and ‘non-responders’ to treatment reported differences in the morphometry and functional connectivity of fronto-striatal, parietal, and cingulo-opercular regions [21–23]. These prior MRI studies were valuable first steps. However, they were only of children and did not investigate the utility of measures of brain anatomical connections as potential factors associated with treatment response in adult ADHD. This is perhaps especially important in conditions—such as ADHD—that are associated with ‘dysconnectivity’ in specific brain circuits.

Anatomical brain connections can be studied using diffusion-weighted imaging, which measures water diffusivity and quantifies the microstructural properties of white matter fibers. Prior diffusion imaging studies in ADHD mainly focused on fronto-striatal networks, in line with the dominant neuropathological hypothesis of this condition. In addition, they provided evidence that other networks may be important, e.g. the fronto-parietal network [24]. The fronto-striatal network (including the fronto-striatal tract and anterior thalamic projections) mediates executive functions, whilst the fronto-parietal network supports distinct aspects of attention and motor control through the three branches of the superior longitudinal fasciculus (SLF I, II, III) [25, 26]. However, the potential independent contribution of the SLF branches to ADHD symptoms—and treatment response—has not been clarified.

Therefore, in this study we used advanced diffusion tractography, based on an algorithm known as spherical deconvolution (SD), to dissect the two main divisions of the fronto-striatal network and the three branches of the SLF. We then tested whether tract metrics were associated with treatment response in adults with ADHD. Finally, as ADHD is a highly heterogeneous condition that is associated with alterations occurring at multiple biological levels, we used a multimodal approach based on machine learning to combine our strongest tract metric with clinical and neuropsychological characteristics and identify a more comprehensive profile of factors associated with treatment response [27, 28].

## Methods

### Sample and research protocol

This study is part of a larger trial employing a single-blind placebo-controlled cross-over design, followed by a longitudinal open-label phase. The trial was registered at ClinicalTrials.gov (NCT 03709940) and conducted at the Maudsley Hospital (London, UK). Please see Supplementary Methods for details on the protocol, power calculation, and inclusion criteria (p. 2). In this study, we specifically tested whether pre-treatment brain anatomy was associated with treatment response in adult ADHD. Sixty male adults with ADHD completed clinical and behavioral measures under placebo (baseline) and under an acute dose of MPH (acute MPH); and underwent diffusion imaging scanning before starting treatment with the same long-acting formulation of MPH (Concerta XL, titrated up to 54 mg). Treatment response was measured at two months (follow-up) (see below). We mainly recruited ADHD medication-naïve participants, and none of the few previously treated had received pharmacological treatment for at least a year prior to this study. At baseline, intelligent quotient (IQ) was measured with the Wechsler Abbreviated Scale of Intelligence (WASI) [29]; and handedness with a modified version of the Edinburgh Handedness Inventory (EHI) [30]. Clinical symptoms were measured using the Barkley Adult ADHD Rating Scale-IV (BAARS-IV) [31]. Behavioral tests included the Quantitative behavior (Qb) test (https://www.qbtech.com), a computer-based test used to measure core ADHD symptoms through a CPT and infrared monitoring of participants’ movements, which was granted approval from the Food and Drug Administration to aid treatment evaluation in individuals with ADHD [32]. The Qb test measures several parameters that are then combined into three Qb scores (Qb activity, Qb impulsivity, and Qb inattention), which represent summary measures of the three cardinal symptoms of ADHD (Supplementary methods, p. 3). ADHD participants also completed urine drug screenings to ascertain abstinence from illicit drugs during the scans, and an MPH assay at follow-up to ascertain treatment adherence. According to previously published criteria, the first definition of treatment response was based on a symptomatic improvement of at least 30% at two months, as measured by the BAARS-IV (total score) [31]. This categorization, although commonly used in clinical practice and in clinical trials, is based on an arbitrary cut-off that might not reflect a biological distinction among participants or apply to a specific sample. For this reason, we also employed an experimental approach based on a data-driven analysis, known as multivariate *k*-mean clustering [33]. This is a common clustering algorithm for multivariate data, which defines whether individuals in the population belong to different groups according to their multiple characteristics. The attribution of individuals to a group is made so that the squared error between the empirical mean of the cluster and the observations in the cluster is minimized [33]. The segregation of our sample into responders and non-responders was based on two clinical variables (reduction of the number of either inattentive or hyperactive/impulsive symptoms from baseline) and three behavioral variables (reduction of the Qb scores for activity, impulsivity, or inattention from baseline). One advantage of this approach is the identification of two more homogeneous subgroups in terms of clinical and behavioral improvement under treatment, which may better reflect, and thus allow us to understand, underlying biological differences among groups, in line with previous reports [34]. Characteristics of the two groups are displayed in Tables S3a-b. Finally, baseline scans were also obtained from 20 neurotypical controls matched for sex, age, and IQ. All participants gave written informed consent before taking part in this research. The authors assert that all procedures contributing to this work comply with the ethical standards of the relevant national and institutional committees on human experimentation and with the Helsinki Declaration of 1975, as revised in 2008. Ethical approval REC number: 12/LO/0630.

### Diffusion MRI: data acquisition and analysis

Scan parameters, pre-processing, and SD tractography are described in Supplementary Methods (p. 3). In brief, preprocessing included correction for susceptibility-induced off-resonance field, motion, and eddy current distortions. We dissected the bilateral fronto-striatal tract, anterior thalamic projection, and three branches of the SLF, as previously described [25, 35]. For each tract we extracted a metric indicative of the size of the tract, i.e. the volume, and a metric reflecting its microstructural organization, i.e. the hindrance modulated orientational anisotropy (HMOA) [36]. We selected this metric, as it represents a compact measure of fiber diffusion properties in SD tractography as FA does in conventional diffusion tensor imaging (DTI) but can be more reliably used in regions with complex fiber organization, such as those crossed by the SLF [36]. Finally, we used volume to calculate the Lateralization Indices (LIs) for each pair of the SLF branches, according to the formula: (Right volume−Left volume)/(Right volume + Left volume). Positive values indicate a rightward asymmetry, whereas negative values reflect a leftward asymmetry. We selected this metric because there is evidence that the pattern of lateralization of the SLF branches is associated with attention performance in neurotypical adults [25]. In line with the Human Brain Mapping Committee on Best Practice in Data Analysis and Sharing (COBIDAS) guidelines on data sharing (https://www.humanbrainmapping.org/files/2016/COBIDASreport.pdf), de-identified raw diffusion imaging data will be made publicly available at http://www.bcblab.com upon publication.

### Statistical analysis

#### Categorical approach

We used SPSS (v26, IBM) to confirm the normality of tract metrics and conduct the statistical analyses. Taking a categorical approach, the association between tract metrics and treatment response was tested using logistic regressions, in which the independent variable was one tract metric (volume, LI, and HMOA for the ten selected tracts) and the dependent variable was treatment response (as classified by either BAARS-IV score or *k*-mean clustering). As a descriptive measure of the association with treatment response, we calculated the area under the curve (AUC) from the corresponding receiver operating characteristic (ROC) curves. We also performed 10-fold cross-validation to estimate the strength of the identified factors associated with treatment response in new data. Family-wise error correction for multiple comparisons (total number of tracts) was performed with a permutation test [37]. We chose this method because we observed relevant correlations between the tracts of interest (Table S7). Clinico-demographic characteristics that could be potential confounders (age, MPH dose, weight, baseline severity, years of previous treatment, and handedness) were afterwards included as covariates.

To better understand the association between tract metrics found significant by logistic regression and treatment response, we conducted these two secondary analyses.

#### Comparison with controls

Independent-sample *t*-tests (two-sided) were performed to determine whether responders and non-responders differed from controls in the tract metrics revealed as significant by logistic regression.

#### Dimensional approach

We used linear regressions to examine whether the association between tract metrics and treatment response was driven by improvement on either inattentive and/or hyperactive/impulsive symptoms (i.e., reduction of BAARS-IV Inattention or Hyperactivity/Impulsivity scores at follow-up as compared to baseline).

### Machine learning

Finally, we explored whether combining our strongest imaging metric with clinical and neuropsychological data through machine learning provided a more comprehensive profile of factors associated with treatment response (classification based on *k*-mean clustering). We included clinico-demographic and neuropsychological variables, as previously suggested [14, 15]. The former included age, ADHD presentation, baseline severity, medication-naïve status, MPH dose at follow-up, treatment adherence, and use of illicit drugs. Neuropsychological variables included handedness, total IQ, and measures of inattention, impulsivity, and hyperactivity as extracted from the CPT during the Qb test, both under placebo and under an acute dose of MPH. Please see Supplementary Methods (p. 3) for the complete list of variables extracted. We created different models using Least Absolute Shrinkage and Selection Operator (LASSO) regressions. These are conceptually very similar to standard multiple logistic regressions, except that they can accept more independent variables, even if collinear, because they automatically select a limited set of them. The lasso regression has a regularization parameter to select more or fewer independent variables. To find the optimal regularization parameter, we conducted 10-fold cross-validation and chose the parameter with the minimum cross-validation error. Due to our relatively small sample size, we used the whole sample to both fit the lasso regression and assess its accuracy. Potential limitations of this approach are presented in the discussion. Before conducting the lasso regression, we used multiple imputations to estimate the missing values [38], creating several imputed datasets. Specifically, we imputed 20 datasets per run [39], and we ran the process 10 times, so we obtained 200 models. Among these models, those including a higher number of variables may achieve a higher balanced accuracy (BAC) but are also more likely to overfit. Hence, to limit overfitting while achieving optimal accuracy, we selected the model with the least number of variables that produced a BAC above 80%, as previously recommended [40].

## Results

### Sample

60 male adults with ADHD completed the study (see Fig. S1 for Flow Diagram). They were mostly White British (71%), had a mean (±SD) age of 28 (±7) years and a full-scale IQ of 109 (±12); the majority was right-handed (78%) and medication-naïve (77%). Data on individuals previously exposed to medication is reported in Table S2. Controls were matched for sex, age, and IQ, and were mostly right-handed (90%). Concerta XL was titrated up to 54 mg in most participants, but the dose had to be reduced to 36 mg in 24% of cases and to 18 mg in 7% of cases due to side effects. Only two participants increased the dose. We considered dose at follow-up among the potential confounding factors in our statistical analysis (see the “Methods” section). At follow-up, 42 participants were identified as responders and 18 as non-responders based on their BAARS-IV score. *K*-mean clustering identified a group with high and a group with low average improvement on BAARS-IV and Qb scores, which were respectively labeled as responders (*N* = 26) and non-responders (*N* = 34) (Tables S3, S4). With either classification, responders, and non-responders did not significantly differ in ethnicity, age, total IQ, handedness, clinical presentation, and MHP dose (Table S5). According to the classification based on a 30% BAARS-IV improvement cut-off, there was no significant difference in baseline symptom severity between groups; however, we found a significant difference when using the classification based on *k*-mean clustering (Table S5). We considered baseline severity among the potential confounding factors in our statistical analysis (see the “Methods” section). Drug screening and MPH assay results are reported in Supplementary results (p. 6).

### Statistical analysis

#### Categorical approach

**Fig. 1:**
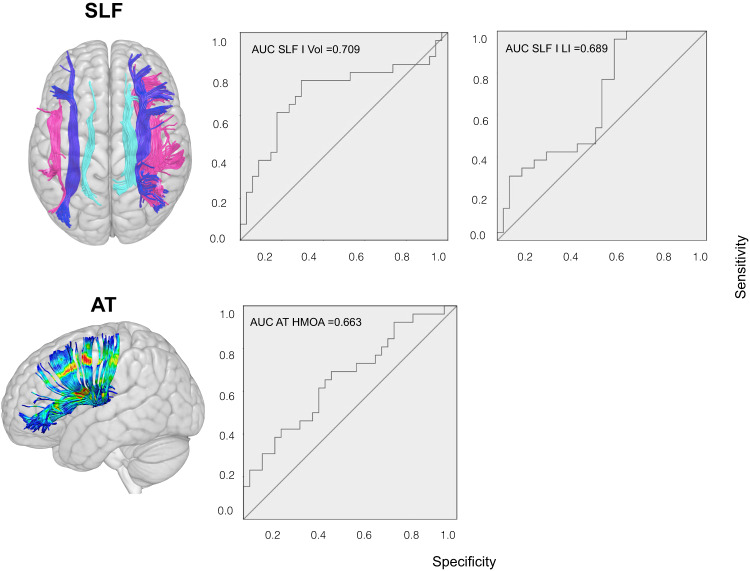
ROC curves. Plots for the SLF I Volume, SLF I lateralization index, and left anterior thalamic HMOA. The left SLF I volume was the strongest associate of treatment response. AT anterior thalamic, AUC area under the curve, HMOA hindrance modulated orientational anisotropy, LI lateralization index, *ROC* receiver operating characteristic, SLF superior longitudinal fasciculus, Vol volume.

We analyzed whether tract metrics were associated with treatment response by using logistic regression. When we tested the previously published definition of response, i.e., symptom reduction of at least 30%, we observed that a smaller left SLF I volume was associated with non-response only at a trend level (Wald’s *X*^2^(1,60) = 3.502, *p* = 0.061). In contrast, when we used the data-driven classification responders/non-responders based on *k*-mean clustering, we found that ADHD participants with a smaller left SLF I volume, increased right-lateralization of the SLF I, or greater left anterior thalamic HMOA had a significantly lower probability of being responders to two months of MPH treatment (Table 1). No associations were detected for the fronto-striatal tract. All logistic regression results are reported in Table S6. ROC curves are presented in Fig. 1. AUCs, specificity, and sensitivity at optimal cut-off are reported in Table 1.

**Table 1:** Tract metrics and treatment response.

TRACT METRIC	LOGISTIC REGRESSION			ROC			FAMILY-WISE CORRECTION		
	*B*	Wald’s *X*^2^	*p*-value	AUC	Sens. (%)	Spec. (%)	Est.	*Z*-value	Corr. *p*-value
Left SLF I volume	0.255	6.955	**0.008**	0.709	76.9	70.6	0.255	2.637	**0.038**
SLF I LI	−3.627	5.254	**0.022**	0.689	96.2	45.5	−3.627	−2.292	0.075
Left AT HMOA	−101.364	4.387	**0.036**	0.663	65.4	61.8	−33.092	−0.692	0.997

The first columns display the statistically significant results of the logistic regression testing the relationship between tract metrics and treatment response (i.e., the classification responders/non-responders based on *k*-mean clustering). The following three columns report AUC, specificity, and sensitivity at optimal cut-off of the corresponding ROC curves. Finally, the last three columns display family-wise correction for multiple comparisons. Statistically significant results are reported in bold. The left SLF I volume was the strongest associate of treatment response and survived correction for multiple comparisons (number of tracts = 10).

*AT* anterior thalamic, *AUC* area under the curve, *B* tract metric regression coefficient, *Corr* corrected, *Est* estimate, *HMOA* hindrance modulated orientational anisotropy, *LI* lateralization index, *ROC* receiver operating characteristic, *Sens* sensitivity at optimal cut-off, *SLF* superior longitudinal fasciculus, *Spec* specificity at optimal cut-off.

The regression including the left SLF I volume survived family-wise correction for multiple comparisons (number of tracts = 10) (Table S8). ROC curve analysis revealed that the left SLF I volume was moderately associated with treatment response (AUC = 70.9%). The 10-fold cross-validation showed that 70% of the new observations kept the association between the left SLF I volume and treatment response (Supplementary results, p. 10). Further, the association between the left SLF I volume and treatment response remained statistically significant after controlling for handedness, years of previous treatment, and MPH dose (all *p* < 0.01), and when controlling for age, baseline severity, and weight (respectively, *p* = 0.011, *p* = 0.012, and *p* = 0.019).

We then investigated the relationship between the three significant tract metrics and treatment response (as classified by *k*-mean clustering) in the following two secondary analyses. Given the exploratory aim of these analyses, and for completeness, we also carried out these analyses for the two metrics that did not survive correction for multiple comparisons (SLF I lateralization index and left anterior thalamic HMOA), although caution should be used when interpreting them.

#### Comparison with controls

**Fig. 2:**
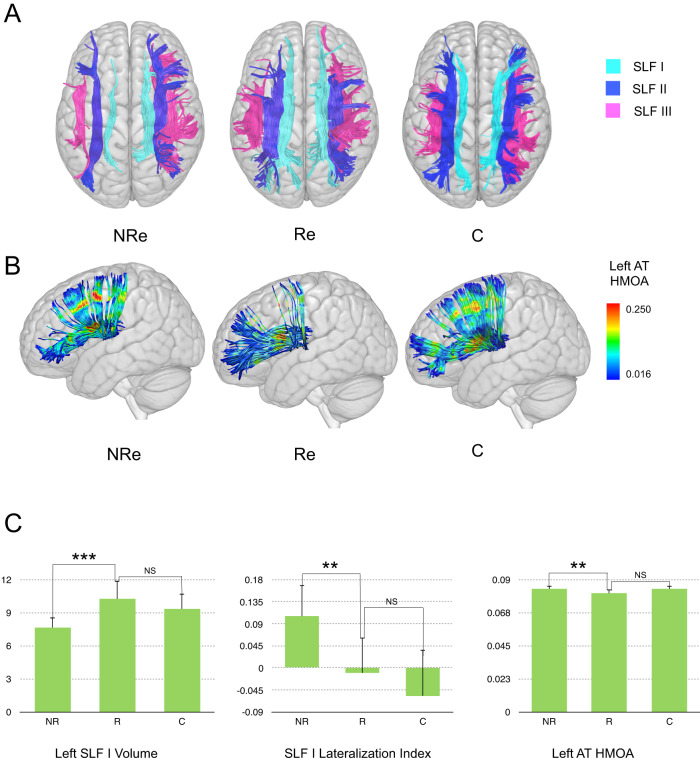
Tract metrics in non-responders (NRe), responders (Re), and controls (C). Panels **A** and **B**, respectively, show the tractography reconstruction of the SLF branches and the left anterior thalamic radiation. As displayed in panel **C**, participants with ADHD with smaller left SLF I volume and greater right-lateralization of the SLF I had a lower probability of being responders to two months of treatment. Notably, responders did not differ from controls in these tract metrics. Further, participants with ADHD with greater left anterior thalamic HMOA had a lower probability of being responders to two months of treatment. Again, responders did not differ from controls in this tract metric. AT anterior thalamic, HMOA hindrance modulated orientational anisotropy, SLF superior longitudinal fasciculus.

Considering the definition of treatment response based on k-mean clustering, non-responders significantly differed from controls in the left SLF I volume (*t*(52) = −2.172, *p* = 0.034), whereas responders did not (*t*(44) = 0.881, *p* = 0.383). As detailed in Supplementary results (p. 10), non-responders also differed from controls in the SLF I lateralization index, whereas responders and controls did not significantly differ in both the SLF lateralization index and the left anterior thalamic HMOA. That is, the anatomy of individuals with ADHD who responded to treatment did not significantly differ from that of controls in all three metrics (Fig. 2).

#### Dimensional approach

**Fig. 3:**
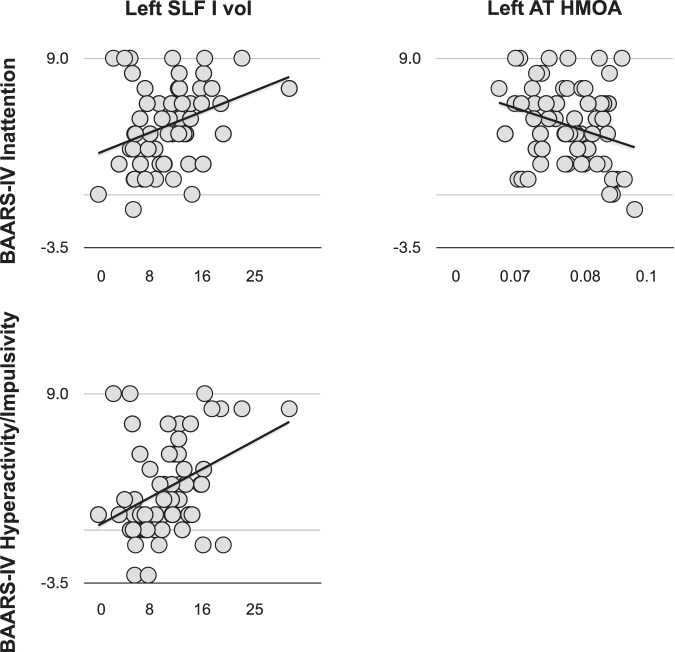
Linear regressions—plots of the statistically significant results. The left SLF I volume was associated with the degree of symptomatic improvement on both inattentive and hyperactive/impulsive symptoms. The left anterior thalamic HMOA was only associated with the improvement on inattentive symptoms. AT anterior thalamic, HMOA hindrance modulated orientational anisotropy, SLF superior longitudinal fasciculus, vol volume.

When we used linear regression to test whether the association between tract metrics and treatment response was driven by improvement on either inattentive and/or hyperactive/impulsive symptoms, we observed that the left SLF I volume was positively associated with the individual degree of improvement on both inattentive and hyperactive/impulsive symptoms (*p* = 0.011 and 0.003, respectively). Further, the left anterior thalamic HMOA was negatively associated with improvement on inattentive symptoms (Table 2 and Fig. 3).

**Table 2:** Linear regressions.

Tract metric	BAARS-INA				BAARS-HI			
	Equation	*F*	*p*	*R*^2^	Equation	*F*	*p*	*R*^2^
Left SLF I volume	SI = 2.340 + .250 SLF I Vol (ml)	6.968	**0.011**	0.107	SI = −0.229 + 0.339 SLF I Vol (ml)	9.717	**0.003**	0.144
SLF I LI	SI = 4.667−2.441 SLF I LI	1.722	0.195	0.029	SI = 2.895−2.640 SLF I LI	1.454	0.233	0.024
Left AT HMOA	SI = 15.137−128.551 AT HMOA	4.899	**0.031**	0.078	SI = 3.804−12.780 AT HMOA	0.032	0.858	0.001

Linear regression revealed that the left SLF I and the left anterior thalamic HMOA were associated with the degree of symptomatic improvement after two months of treatment (i.e., reduction of the number of symptoms from baseline). Symptom improvement at follow-up can be calculated according to the formula *Y* = *α* + *βX*, where *α* and *β* are the regression coefficients and *X* is the tract metric of interest. Statistically significant results are displayed in bold.

*AT* anterior thalamic, *BAARS-HI* Barkley scale-hyperactive/impulsive symptoms, *BAARS-INA* Barkley scale-inattentive symptoms, *HMOA* hindrance modulated orientational anisotropy, *LI* lateralization index, *SI* symptom improvement, *SLF* superior longitudinal fasciculus, *Vol* volume.

### Machine learning

Finally, lasso regression produced 200 treatment response models with an average BAC of 96.6%. To limit overfitting while achieving optimal accuracy, we selected the model with the least number of variables that showed at least 80% BAC [40]. The chosen model achieved 82.5% BAC (as compared to 73.7% for the left SLF I volume alone) and included 10 variables: left SLF I volume, baseline severity of inattentive and hyperactive/impulsive symptoms, medication-naïve status, MPH dose, handedness (categorical and dimensional score), total IQ, Qb impulsivity score after an acute dose of MPH, and baseline Longest Passivity (i.e., the maximum number of consecutive omission errors at the CPT as measured by the Qb test). The exponentiated regression coefficients (odds ratios) for each included variable are reported in Table S9.

## Discussion

For the first time, we examined the relationship between the anatomy of specific brain networks and response to MPH in adults with ADHD. Our results suggest that a smaller left SLF I, which corresponds to the dorsal attentive network (DAN) [41], was associated at a group level with a significantly lower probability of being responders to two months of MPH treatment. Secondary analyses indicated that the association between the left SLF I volume and treatment response was driven by improvement on both inattentive and hyperactive/impulsive symptoms and that only non-responders significantly differed from controls in this tract metric. Overall, these novel findings suggest that a smaller left DAN may contribute to the pathophysiology of adult ADHD and partly explain variation in treatment response.

The fact that the SLF I is related to treatment response is likely explained by its role in supporting core brain functions implicated in ADHD, such as attention, working memory, and motor control [25, 26, 41–43]. Further, prior diffusion imaging studies identified alterations in the SLF of participants with ADHD as compared to controls, which were associated with symptom severity and neuropsychological deficits [44–49]. Only a few imaging studies reported differences in fronto-parietal networks between responders and non-responders and were all in children [22, 23], although a recent small study reported improvement of visuo-spatial attention under MPH in children with a dorso-ventral gradient of the FA of the SLF [50]. We added to these prior findings as we observed that only the SLF I was associated with treatment response in adults, and that only non-responders significantly differed in this metric as compared to controls. Our results suggest that some anatomical alterations may be more evident in non-responders and may contribute to their poor treatment response.

Unfortunately, tractography does not allow the direct investigation of the biological mechanisms through which a smaller left SLF I might influence treatment response. A smaller tract may be related to altered white matter (e.g. myelination) or gray matter (e.g. reduced number or size of axons) [36], both of which have been previously suggested as pathophysiological mechanisms in ADHD [23, 51–53]. Ultimately, a smaller tract may affect conduction speed and thus brain function [54, 55]. We might speculate that an asymmetrical representation of the SLF I in the two hemispheres may condition an imbalance between hemispheric contributions during the voluntary control of attention and may lead to less efficient suppression of the right-lateralized ventral attentive network (VAN), which in turn may enhance distractibility [56]. This imbalance might also potentially hinder MPH modulatory activity on these networks. We therefore encourage future studies to investigate the role of asymmetry of attentive networks in ADHD pathophysiology. Although the underlying mechanisms are unknown, our results are in line with animal studies suggesting that therapeutic doses of stimulants mainly affect catecholamine levels at a cortical level [57, 58]; and with prior imaging studies reporting stimulant-related functional changes in frontoparietal networks in adults with ADHD [59–61].

Nevertheless, as supported by our machine learning approach, other individual characteristics, such as clinical and cognitive profiles, likely also play a role in treatment response [14, 15]. For instance, we observed that a lower total IQ and more severe inattention and impulsivity levels at the CPT (at baseline or under an acute dose of MPH, respectively) were associated with worse response. This was also observed in some previous reports [16, 17], although not all confirmed the potential association of IQ with outcome [14]. As prior studies suggested that executive attention might play an important role in intelligence, acting as a bottleneck on task performance [62], it is possible to speculate that poorer attentive functions may be associated with both a lower total IQ and lower responsiveness to MPH. However, further studies are needed to clarify how general cognitive levels may be linked to treatment response. Further, left-handedness appeared to be indirectly associated with poorer response. Prior studies reported conflicting results on handedness and ADHD, with findings varying from normal to higher rates of left-handed subjects among participants with ADHD [63, 64]. We found a higher proportion of left-handed subjects in our ADHD group as compared to controls and this, together with the neuropsychological findings from the studies cited above, may further support the suggestion that response to stimulants is affected by the brain anatomical and functional organization on which MPH acts. Finally, clinical characteristics such as baseline symptom severity, medication-naïve status, and MPH dose may also be important—in agreement with previous clinical studies [14, 65, 66]. Taken together, these findings suggest that, given the heterogeneity of ADHD, a profile that combines information by integrating data from multiple sources may be more effective in identifying factors associated with treatment response.

Overall, this study is one of the largest diffusion imaging studies in adult ADHD and benefitted from the longitudinal design and the use of an advanced tractography method; however, limitations should be taken into consideration. We included a small percentage of participants previously treated with ADHD medication. Exposure to medication has been suggested to have a ‘normalizing’ effect on brain structure by some prior studies [67], although more recent reports suggest that this may not be the case [68]. Our findings suggest that the observed ‘normalizing’ effect of long-term treatment on brain anatomy may be at least in part dependent on a selection bias, as responders to treatment have fewer pre-treatment brain alterations. Further, this study was designed as an open trial, as we were unable to carry out a randomized controlled trial due to ethical constraints—as this would mean withholding what is an effective treatment. Nonetheless this is unlikely to have affected our results because virtual dissections were performed using a semi-automated procedure and blind to-group; and the symptom rating scale was integrated with objective measures of treatment response, such as the Qb scores. Finally, we only recruited males, because the prevalence of ADHD has been reported to be more than double in males as compared to females [69], and there is preliminary evidence of sex differences in brain connectivity [70, 71]. However, it is not known whether sex differences in brain connectivity may relate to sex differences in treatment response. Thus, to avoid potential sex-related confounding on our connectivity analyses, we only included males, but we encourage future studies to extend our analyses to the female ADHD population. Similarly, we only included participants without comorbid conditions because there is evidence that differences in brain connectivity and treatment response may exist between individuals with/without comorbidities [72]. For instance, recent meta-analyses highlighted both shared and specific connectivity alterations in individuals with ADHD and autism spectrum disorder (ASD) [73, 74]; and individuals with both conditions have lower response rates to stimulants [75]. Considering that this is the first study investigating the association between anatomical connectivity and treatment response in adult ADHD, we wanted to avoid potential comorbidity-related confounding. However, our results should be validated in clinical samples including individuals with comorbidities.

Investigating factors associated with treatment response represents a first step towards the identification of potential predictors of treatment response [13]. This is of relevance because, although at a group level MPH has been proven to be effective in ameliorating symptoms, there is individual variability in response, and this affects outcomes [8]. Further, the choice of medication in clinical practice is based on a trial-and-error process. Thus, there is increasing recognition that we need to better understand the biological basis of treatment response and (where possible) at an individual level [13]. However, to be tested as potential predictors according to the principles of precision psychiatry, the anatomical factors associated with treatment response that we report should first be replicated in independent samples and, preferably, confirmed by meta-analyses [76]. Further, although machine learning approaches, like the one we used in our exploratory analysis, have been deemed to be suitable to combine variables at multiple interacting levels, and we performed 10-fold cross-validation as a means of ‘internal’ validation, we did not have an independent sample for external validation, which might have led to inflated accuracy. Therefore, our results need to be validated in an external sample—and of sufficient size to ensure stable and generalizable accuracy estimates [76]. Only once these steps have been completed, can the utility of our proposed model be tested in pragmatic randomized trials [13, 77]. Nevertheless, identifying factors associated with treatment response is a necessary and valuable first step.

In conclusion, we provided novel evidence that the anatomy of the DAN may partly explain variation in treatment response in adult ADHD. If replicated, these findings may help identify predictors of treatment response and guide the development of new treatments.

### Supplementary information

Supplementary material
