# Supplementary material for: Poor response to methylphenidate is associated with a smaller dorsal attentive network in adult Attention-Deficit/Hyperactivity Disorder (ADHD)
Source: Transl Psychiatry. 2023 Sep 30;13:303. doi: 10.1038/s41398-023-02598-w (PMC10542768; doi:10.1038/s41398-023-02598-w)
Supplement: Supplementary file 1 — Supplementary material [file 41398_2023_2598_MOESM1_ESM.docx]

**SUPPLEMENTARY MATERIAL**

**TABLE OF CONTENT**

**SUPPLEMENTARY METHODS page 2**

*Power calculation and inclusion criteria page 2*

*Research protocol page 2*

*Qb test page 3*

*Diffusion MRI data acquisition, preprocessing and tractography page 3*

**SUPPLEMENTARY RESULTS page 5**

*Study flow diagram page 5*

*Additional sample characteristics page 6*

*Treatment response: categorical classification page 7*

*Main statistical analysis page 8*

*Cross-validation page 10*

*Group comparison page 10*

*Lasso regression page 11*

***FIGURE AND TABLES***

*Figure S1. Study Flow Diagram page 5*

*Table S1. Composition of Qb scores page 3*

*Table S2* *Individuals previously exposed to ADHD medication page 6*

*Table S3a. Classification Responders/Non-responders based on BAARS-IV score page 7*

*Table S3b. Classification Responders/Non-responders based on k-mean clustering page 7*

*Table S4. Correlations between improvement variables page 7*

*Table S5. Demographic and baseline clinical characteristics according to group page 8*

*Table S6. Binary logistic regression page 8*

*Table S7. Correlations among tract metrics significant at the logistic regression page 9*

*Table S8. Family-wise correction for multiple comparisons page 9*

*Table S9. Results of the lasso regression page 11*

**REFERENCES page 11**

**SUPPLEMENTARY METHODS**

***Power calculation and inclusion criteria***

We recruited 60 male adults with Attention-Deficit/Hyperactivity Disorder (ADHD) among those referred for an assessment to the Adult ADHD Clinic, Maudsley Hospital, London. The sample size was determined based on a power calculation: considering an effect size d=0.04 (SD=0.05)([1](#_ENREF_1)), 60 ADHD adults were necessary to obtain 20 (34% of 60) participants for which the treatment was ineffective ([2](#_ENREF_2)), with a statistical power above 80%. Participants were deemed eligible if they fulfilled the following inclusion criteria: ADHD diagnosis (confirmed by a clinician according to the DSM-V criteria), non-medicated, aged 18-45 years old, with an intelligence quotient (IQ) above 70, and no current clinically diagnosed comorbid disorder or medical condition precluding magnetic resonance imaging (MRI) scanning.

The diagnosis of ADHD was confirmed by a clinician of the Maudsley Hospital following psychiatric assessment and the *Diagnostic Interview for ADHD in Adults (DIVA) 2.0. (*[*3*](#_ENREF_3)*)*. This semi-structured interview was originally developed to diagnose adult ADHD according to the DSM-IV criteria, but we applied the diagnostic threshold updated by the DSM-V, which considers five symptoms (instead of six) as sufficient for a reliable diagnosis in adulthood.

Regarding psychiatric comorbidity, subclinical anxiety or depressive symptoms were allowed, as well as social alcohol consumption (but not alcohol dependence). None of the participants was on treatment with any psychotropic medication at the time of the study. Occasional use of recreational drugs is very common in ADHD and was not considered an exclusion criterion. However, all participants underwent urine drug screening to ascertain that they were abstinent from substances during the study (see results).

***Research protocol***

This study is part of a larger clinical trial employing a single-blind placebo-controlled cross-over design, followed by a longitudinal open-label phase (NCT 03709940). In brief, the study was developed over three sessions, two at baseline (DAY 1 and DAY 2) and one after two months of treatment with methylphenidate (MPH) (follow-up).

The first two sessions were conceived as a single-blind non-randomized placebo-controlled cross-over experiment. The first 30 subjects received a placebo tablet (ascorbic acid 50 mgs) on DAY 1 before the behavioral assessment (Qb test) and MRI scan. The behavioral assessment and part of MRI measurements (functional MRI and resting state functional connectivity) were repeated two days after (DAY 2), under a clinically effective dose of short-acting MPH (20 mgs). The order of the tablets was reverted for the remaining 30 participants to balance any potential expectation and practice effect between the two conditions. Hence, the tablets were administered in a prefixed non-randomized order. The study was not in fact conceptualized as a clinical randomized trial, aiming at the comparison between placebo and medication effects, for which the lack of randomization would be a major confound. It instead investigated whether an acute response to medication was associated with post-titration treatment response. The MPH dose used (20 mgs) was slightly above that recommended by the NICE guidelines ([www.nice.org.uk](http://www.nice.org.uk)) as a starting dose (15 mgs/day), as this was shown to affect brain activation during fMRI tasks in adults ([4](#_ENREF_4)). Behavioral tests started 1 hour after drug administration and the scan 3 hours after. As the maximum plasma concentration (Cmax) after 20 mgs of MPH is attained on average 90 min after administration, with a range between one to two hours ([4](#_ENREF_4)), the set timing allowed participants to perform behavioral tests and functional MRI acquisitions under an optimal dose of MPH. The protocol followed during the two sessions was identical in respect of timing and tests administered to keep the participants blind to the drug condition (medication or placebo).

All participants were then started on the same long-acting formulation of MPH (Concerta XL) (open-label phase), to increase sample homogeneity and promote adherence. The dose was gradually titrated up to 54 mgs. During this time, telephone follow-up appointments were offered to monitor potential side effects and to adjust the dose if needed. We considered dose at follow-up among the potential confounding factors in our statistical analysis. Of note, as illicit drug use and non-adherence to treatment may represent confounding factors, ADHD participants underwent urine drug screening before brain imaging and completed an MPH assay at follow-up to confirm adherence to treatment. Finally, participants’ data from the Qb test was extracted by an independent researcher blind to group, and imaging data analysis was performed using a semi-automated procedure and blind to group.

***Qb test***

The Qb test (<https://www.qbtech.com>) is a computer-based assessment tool that measures several parameters, e.g., time active, omission and commission errors, while the participant is performing a continuous performance task (CPT), and provides three summary measures (Qb activity, Qb impulsivity and Qb inattention) (Table S1).

**Table S1. Composition of Qb scores.**

| **QB SCORE** | **Parameters** |
| --- | --- |
| Qb Activity | **Time active** (i.e., time in per cent a participant moves more than 1cm/sec);  **Distance** (i.e., distance covered by the marker in meters);  **Area** (i.e., area covered by the marker);  **Micro-events** (i.e., how many times a participant moves more than 1mm). |
| Qb Impulsivity | **Commission errors** (i.e., the number of responses to non-target stimuli);  **Normalized commission errors** (i.e., the ratio of commission errors to the number of corrected responses). |
| Qb Inattention | **Omission errors** (i.e., the number of missed responses);  **Average reaction time**;  **Reaction time variability** (i.e., the standard deviation of reaction time). |

For completeness, in the machine learning data analysis, we considered the three Qb scores (under placebo and under an acute dose of MPH); the individual parameters listed in table S1; and the following additional parameters: Simplicity (i.e., complexity of the movement path of the reflective marker); Error rate (i.e., frequency of incorrect responses); Dprime (i.e., measure of the participant’s ability to distinguish between Targets and Non-targets); and Longpass (i.e., Longest Passivity or the maximum number of consecutive Omission Errors).

***Diffusion MRI data acquisition, preprocessing and tractography***

Diffusion imaging data was acquired on a 3T MR750 GE scanner (General Electric, Milwaukee, WI, USA), using a spin-echo EPI sequence, with these parameters: 72 slices, voxel size 2x2x2 mm, matrix 128x128, TE 79.8 ms, TR 12.000 ms, flip angle 90°, b-value 1500 s/mm^2^, 60 diffusion-weighted directions and six non-diffusion-weighted volumes. Peripheral cardiac gating was applied to avoid artefacts due to brain pulsation.

‘Top-up’ was used as implemented in FSL ([www.fmrib.ox.ac.uk/fsl](http://www.fmrib.ox.ac.uk/fsl))([5](#_ENREF_5)) to correct for susceptibility-induced off-resonance field distortions ([6](#_ENREF_6)); and was followed by correction for motion and eddy current distortions, through iterative affine registration to the six non-diffusion weighted volumes ([7](#_ENREF_7)), as implemented in ExploreDTI (www.exploredti.org). No participant had to be excluded after visual inspection of data quality.

Spherical deconvolution (SD) with a damped Richardson-Lucy (dRL) algorithm was used to estimate white matter fiber orientations, as the investigated regions contain distinct fiber populations, such as crossing fibers ([8](#_ENREF_8)) ([9](#_ENREF_9)). Estimates of fiber orientation were obtained by identifying the local maxima of the corresponding peaks of the fiber orientation distribution. An absolute threshold, corresponding to the threefold amplitude of a fiber orientation distribution derived from a grey matter isotropic voxel, was applied to exclude small local maxima related to isotropic tissue or noise. Further, a relative threshold, corresponding to the 5% of the maximum fiber distribution amplitude, was applied to remove local maxima with values above the absolute threshold ([8](#_ENREF_8)). Streamlines were then reconstructed by using a modified Euler tractography algorithm following subsequent estimates of fiber orientation voxel by voxel with a step size of 1 mm ([9](#_ENREF_9)). The orientation vector of least curvature was followed in case of voxels containing crossing fibers ([10](#_ENREF_10)). Tracking was halted when the curvature between two steps was above 35°, or when encountering a voxel without fiber orientation. SD modelling and tractography was performed using Startrack (<http://www.mr-startrack.com>).

Tracts were visualized using the software Trackvis (http://www.trackvis.org)([11](#_ENREF_11)). The different components of the fronto-parietal network (three branches of the superior longitudinal fasciculus, SLF) and of the fronto-striatal network were isolated, as previously described ([1](#_ENREF_1), [12](#_ENREF_12)). We used a semi-automatic approach for the dissections to reduce variability ([1](#_ENREF_1), [12](#_ENREF_12)), and subsequently all tracts were inspected by an anatomically trained operator for the removal of artefacts. The operator was also blind to participants’ response status.

**SUPPLEMENTARY RESULTS**

***Study Flow diagram***

We recruited male adults with ADHD among those referred for an assessment to the Adult ADHD Clinic, Maudsley Hospital, London. 186 participants were initially identified as potentially suitable through screening of referrals between May 2013 and September 2014. Eligible subjects were invited to take part into research once their ADHD diagnosis was confirmed by a clinician of the Maudsley Hospital. As planned, the trial ended when the 60^th^ participant completed the follow-up visit.

**Study flow diagram**


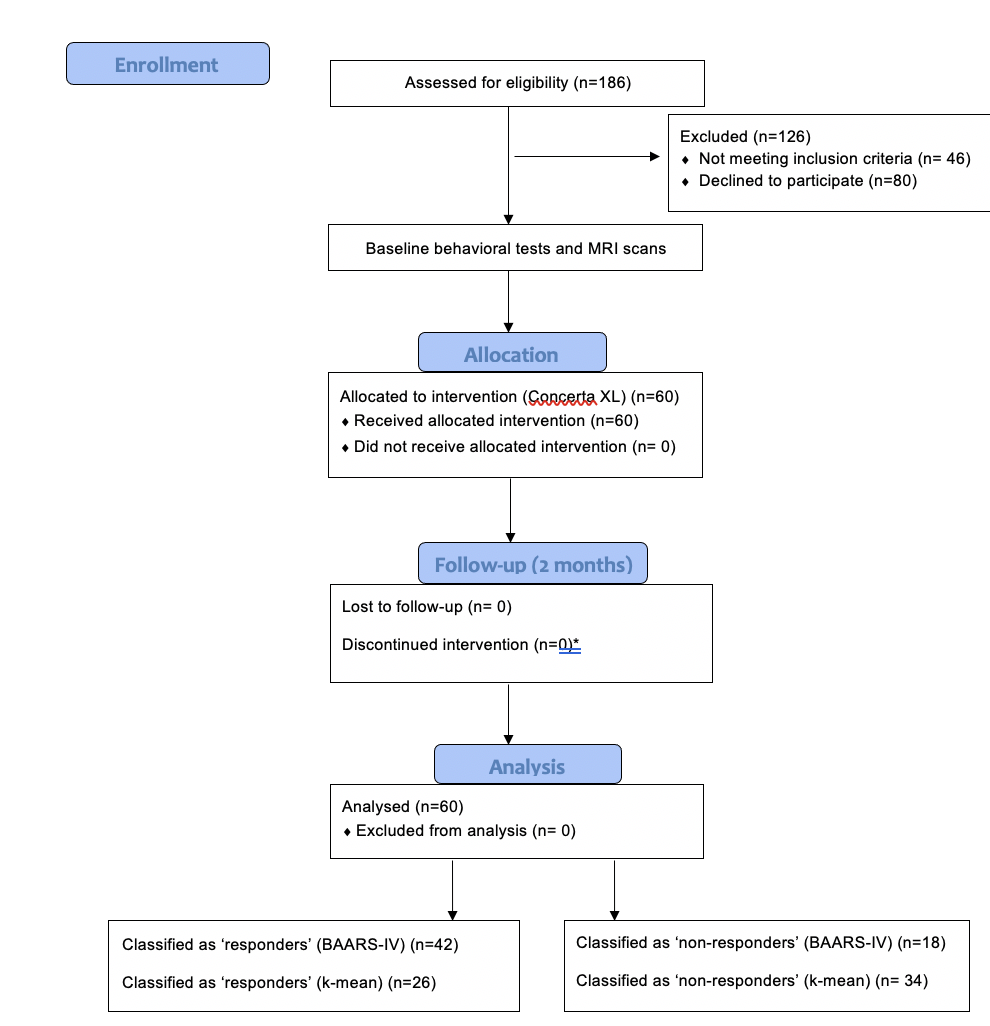


**Figure S1. Study Flow Diagram.** 60 male adults with ADHD completed the study. *= However, MPH assay was negative at follow-up for 6 of the 55 available samples. All 60 patients were analysed in the main analysis (intention to treat approach), and MPH assay results were considered in secondary analysis.

***Additional sample characteristics***

The great majority of participants (72%) was not assessed for ADHD prior to this study. None had a clinically diagnosed co-occurrent condition; however, one of them had received a diagnosis of Oppositional Defiant Disorder (ODD) and 8 of Dyslexia as a child. Most participants were ADHD medication-naïve (77%); and none was taking psychotropic medication at the time of the study. Among the 14 individuals that were previously treated with ADHD medication, only three were classified as responders during the current study (Table S2). Out of the 56 completed urine drug screenings, 46 were negative (82%); and out of the 55 completed MPH assays, 49 were positive at follow-up (89%). All 60 participants were analyzed in the main analysis using an intention to treat approach. However, medication-naïve status, treatment adherence, and use of illicit drugs were considered as covariates in the lasso regression.

**Table S2. Individuals previously exposed to ADHD medication.** 14 individuals with ADHD were previously exposed to ADHD medication (N=14). On average pharmacological treatment lasted 5 years (range 2 months - 8 years) and was interrupted 8 years before the current study (range 1 - 18 years). Among these 14 individuals, only three were classified as responders during the current study.

| Participant | **Previous  Medication** | **Duration of  previous  treatment** | **How long previous treatment  was stopped for** | **Reason why  treatment was stopped** | **Treatment response during the current study (K-mean)** |
| --- | --- | --- | --- | --- | --- |
|  |  | **(Years)** | **(Years)** |  |  |
| 1 | Methylphenidate short-acting (Ritalin) | 3 | 11 | Side effects | Non-responder |
| 2 | Methylphenidate short-acting (Ritalin) | 0.16 | 4 | Participant was just prescribed a two-month course of medication | Non-responder |
| 3 | Atomoxetine (Strattera) | 8 | 3 | Participant’s own decision | Non-responder |
| 4 | Dexamphetamine | 2 | 17 | Participant’s own decision | Non-responder |
| 5 | Methylphenidate short-acting (Ritalin) | 8 | 18 | Participant’s own decision | Non-responder |
| 6 | Methylphenidate short-acting (Ritalin) | 6 | 5 | Participant’s own decision | Non-responder |
| 7 | Methylphenidate short-acting (Ritalin) | 8 | 9 | Not optimal effect | Responder |
| 8 | Methylphenidate long-acting (Concerta XL) | 2 | 9 | Participant’s own decision | Non-responder |
| 9 | Methylphenidate long-acting (Concerta XL) | 3 | 9 | Participant’s own decision | Responder |
| 10 | Methylphenidate short-acting (Ritalin) | 4 | 12 | Participant’s own decision | Non-responder |
| 11 | Atomoxetine (Strattera). Had no response to stimulants until he tried methylphenidate long-acting (Concerta XL) at high dose (108 mg) | 8 | 1 | Participant’s own decision | Non-responder to 108 mg |
| 12 | Methylphenidate short-acting (Ritalin) | 8 | 3 | Participant’s own decision | Non-responder |
| 13 | Methylphenidate long-acting (Concerta XL) | 4 | 8 | Participant’s own decision | Non-responder |
| 14 | Methylphenidate short-acting (Ritalin) | 7 | 7 | Participant’s own decision | Responder |

***Treatment response: categorical classification***

We used two measures of treatment response, one based on the total BAARS-IV scores, and one based on the integration of the BAARS-IV (inattentive and impulsive/hyperactive symptoms) with the Qb test scores (Qb activity, Qb impulsivity and Qb inattention) as obtained through multivariate k-mean clustering. Tables S2-3 show the composition of the two clinical groups, responders and non-responders, according to the two classification systems. Table S4 displays the relationship between clinical and behavioral variables. Table S5 shows demographic and baseline clinical characteristics by group.

**Table S3a. Classification Responders/Non-responders based on BAARS-IV score.** This table reports data on symptomatic reduction observed at follow-up (compared to baseline) in responders and non-responders, as classified by their total BAARS-IV score (reduction above or below 30%). Average improvement of symptoms and standard deviations are reported.

| **Improvement**  **variable** | **Responders**  **(N=42)** | **Non-responders**  **(N=18)** |
| --- | --- | --- |
| BAARS-IV Total score | 19.90 ± 7.855 | 4.17 ± 4.176 *** |

***= significant group difference as measured by independent sample t-tests (p<.001).

**Table S3b. Classification Responders/Non-responders based on k-mean clustering.** This table reports symptomatic reduction and behavioral improvement measured at follow-up (compared to baseline) in responders and non-responders as classified by k-mean clustering.

| **Improvement**  **variable** | **Responders**  **(N=26)** | **Non-responders**  **(N=34)** |
| --- | --- | --- |
| BAARS-INA | 6.65 ± 1.788 | 2.91 ± 2.080 *** |
| BAARS-HI | 5.54 ± 2.453 | .62 ± 1.596*** |
| Qb activity | 1.519 ± 1.179 | .976 ± 1.043 |
| Qb impulsivity | .958 ± 1.026 | .441 ± .901* |
| Qb inattention | .500 ± 1.035 | .529 ± .976 |

BAARS-INA= Barkley Adult ADHD Rating Scale-IV -inattentive symptoms

BAARS-IH= Barkley Adult ADHD Rating Scale-IV -impulsive/hyperactive symptoms

Asterisks indicate significant group differences as measured by independent sample t-tests (*=p<0.05, ***=p<0.001). Of note the difference for Qb activity was at a trend level (p=0.06).

**Table S4. Correlations between improvement variables.** The improvement of inattentive symptoms was correlated with the improvement of hyperactive/impulsive symptoms, whereas the improvement of Qb activity was correlated with the improvement of the two other Qb scores. Of note, there was no significant correlation between clinical and behavioral outcome variables. Correlation coefficients and p-values are reported. Significant results are reported in bold.

|  | BAARS-INA | BAARS-HI | Qb  activity | Qb impulsivity | Qb inattention |
| --- | --- | --- | --- | --- | --- |
| BAARS-INA | 1 | **r=.556**  **(p=.000)** | r=.161  (p=.220) | r=.137  (p=.295) | r=-.003  (p=.983) |
| BAARS-HI | **r=.556**  **(p=.000)** | 1 | r=.085  (p=.517) | r=.023  (p=.860) | r=-.088  (p=.506) |
| Qb  activity | r=.161  (p=.220) | r=.085  (p=.517) | 1 | **r=.385**  **(p=.002)** | **r=.267**  **(p=.039)** |
| Qb impulsivity | r=.137  (p=.295) | r=.023  (p=.860) | **r=.385**  **(p=.002)** | 1 | r=.025  (p=.848) |
| Qb inattention | r=-.003  (p=.983) | r=-.088  (p=.506) | **r=.267**  **(p=.039)** | r=.025  (p=.848) | 1 |

BAARS-INA= Barkley Adult ADHD Rating Scale-IV -inattentive symptoms

BAARS-IH= Barkley Adult ADHD Rating Scale-IV -impulsive/hyperactive symptoms

**Table S5. Demographic and baseline clinical characteristics according to group.** Responders and non-responders (either classified according to the BAARS-IV score or k-mean clustering) did not significantly differ in ethnicity, age, total IQ, handedness, clinical presentation and MHP dose. Significant results are in bold.

|  | **Responders (BAARS-IV)**  **N=42** | **Non-responders (BAARS-IV) N=18** | **Statistics**  **(t-test or X^2^)** | **Responders**  **(k-mean)**  **N=26** | **Non-responders**  **(k-mean) N=34** | **Statistics**  **(t-test or X^2^)** |
| --- | --- | --- | --- | --- | --- | --- |
| **Ethnicity** N (%)  White British  White (other)  Black British  Black (other)  Asian | 31 (73.8%)  7 (16.6%)  0  1 (2.3%)  3 (7.1%) | 12 (66.6%)  4 (22.2%)  2 (11.1%)  0  0 | X^2^(4)=.6.683 p=.154 | 18 (69.2%)  6 (23%)  0  1 (3.8%)  1 (3.8%) | 25 (73.5%)  5 (14.7%)  2 (5.8%)  0  2 (5.8%) | X^2^(4)=.3.560, p=.469 |
| **Age**  (mean, SD) | 28.6 (7.5) | 27 (6.8) | t(58)=-.752, p=.455 | 29.7 (7.7) | 26.9 (6.9) | t(58)=-1.508, p=.137 |
| **Total IQ**  (mean, SD) | 110.7 (11.8) | 108.1 (13.7) | t(58)=-.736, p=.465 | 113.1 (9.4) | 107.5 (13.8) | t(58)=-1.802, p=.077 |
| **Handedness score**  (mean, SD) | 33.9 (6.9) | 31.5 (8.7) | t(58)=-1.143, p=.258 | 35.1 (5.9) | 31.7 (8.3) | t(58)=-1.740, p=.087 |
| **Baseline BAARS-IV score**  (mean, SD) | 35.48 (10.069) | 34.17 (10.495) | t(58)=-.456,p=.650 | 38.23 (8.604) | 32.68 (10.654) | t(58)=-2.170, **p=.034** |
| **Clinical presentation** N (%)  Inattentive  Combined | 19 (45.2%)  23 (54.7%) | 6 (33.3%)  12 (66.6%) | X^2^(1)=.735 p=.391 | 10 (38.4%)  16 (61.5%) | 15 (44.1%)  19 (55.8%) | X^2^(1)=.194, p=.660 |
| **MPH dose at follow-up (mg)** | 46.29 (12.66) | 49 (17.24) | t(58)=.680  p=.499 | 44.31(14.6) | 49.24 (13.52) | t(58)=1.351  p=0.182 |

BAARS-IV= Barkley Adult ADHD Rating Scale-IV; MPH=methylphenidate

***Main statistical analysis***

Taking a categorical approach, the relationship between tract metrics and treatment response was tested using logistic regression, in which the independent variable was one tract metric (volume, HMOA or lateralization index for the 10 tracts of interest) and the dependent variable was treatment response (as defined by either BAARS-IV score or k-mean clustering) (Table S6). We observed relevant correlations between the tracts of interest (Table S7) and performed a family-wise error correction for multiple comparisons (total number of tracts) with a permutation test (Table S8).

**Table S6. Binary logistic regression.** This table reports Wald’s X^2^ and p-values for all tract metrics based on BAARS-IV score and k-mean clustering. Significant results are shown in bold. Correction for multiple comparisons is reported in Table S7.

| **Tract** | **Metric** | **BAARS-IV Total**  **score** | **k-mean**  **clustering** |
| --- | --- | --- | --- |
| Left SLF 1 | Volume | X^2^=(1,60)=3.502  p=.061 | **X^2^=(1,60)=6.955**  **p=.008** |
|  | HMOA | X^2^=(1,60)=.391  p=.532 | X^2^=(1,60)=.472  p=.492 |
| Right SLF 1 | Volume | X^2^=(1,60)=1.171  p=.279 | X^2^=(1,60)=.434  p=.510 |
|  | HMOA | X^2^=(1,60)=.785  p=.376 | X^2^=(1,60)=.235  p=.628 |
| Left SLF 2 | Volume | X^2^=(1,60)=.725  p=.394 | X^2^=(1,60)=.052  p=.819 |
|  | HMOA | X^2^=(1,60)=.239  p=.625 | X^2^=(1,60)=1.387  p=.239 |
| Right SLF 2 | Volume | X^2^=(1,60)=1.411  p=.235 | X^2^=(1,60)=2.033  p=.154 |
|  | HMOA | X^2^=(1,60)=1.669  p=.196 | X^2^=(1,60)= .185  p=.667 |
| Left SLF 3 | Volume | X^2^=(1,60)=.467  p=.494 | X^2^=(1,60)= 3.160  p=.075 |
|  | HMOA | X^2^=(1,60)=.132  p=.716 | X^2^=(1,60)= .132  p=.717 |
| Right SLF 3 | Volume | X^2^=(1,60)=.477  p=.490 | X^2^=(1,60)= .313  p=.576 |
|  | HMOA | X^2^=(1,60)=1.023  p=.312 | X^2^=(1,60)= .095  p=.758 |
| Left AT | Volume | X^2^=(1,60)=.024  p=.878 | X^2^=(1,60)= 2.661  p=.103 |
|  | HMOA | X^2^=(1,60)=2.334  p=.127 | **X^2^=(1,60)=4.388**  **p=.036** |
| Right AT | Volume | X^2^=(1,60)=.451  p=.502 | X^2^=(1,60)= .005  p=.941 |
|  | HMOA | X^2^=(1,60)=.001  p=.982 | X^2^=(1,60)=.535  p=.464 |
| Left FS | Volume | X^2^=(1,60)=.217  p=.642 | X^2^=(1,60)= .126  p=.723 |
|  | HMOA | X^2^=(1,60)=.002  p=.967 | X^2^=(1,60)= .479  p=.489 |
| Right FS | Volume | X^2^=(1,60)=1.540  p=.215 | X^2^=(1,60)=.365  p=.545 |
|  | HMOA | X^2^=(1,60)=1.184  p=.277 | X^2^=(1,60)=.002  p=.961 |
| **Lateralization index LI=(R-L)/(R+L)** | | | |
| SLF 1 LI | | X^2^=(1,60)=.943  p=.331 | **X^2^=(1,60)=5.254**  **p=.022** |
| SLF 2 LI | | X^2^=(1,60)=2.546  p=.111 | X^2^=(1,60)=1.476  p=.224 |
| SLF 3 LI | | X^2^=(1,60)=.052  p=.820 | X^2^=(1,60)=2.330  p=.127 |
| AT LI | | X^2^=(1,60)=1.897  p=.168 | X^2^=(1,60)=3.213  p=.073 |
| FS LI | | X^2^=(1,60)=1.140  p=.286 | X^2^=(1,60)=.131  p=.718 |

**Table S7. Correlations among tract metrics that were statistically significant at the logistic regression.** Correlation coefficients and p-values are reported. Significant results are highlighted in bold.

|  | Left SLF 1 volume | SLF 1_LI | Left AT HMOA |
| --- | --- | --- | --- |
| Left SLF 1 volume | 1 | **-.257 (0.47)** | **-.312 (.015)** |
| SLF 1 LI | **-.257 (0.47)** | 1 | .174 (.183) |
| Left AT HMOA | **-.312 (.015)** | .174 (.183) | 1 |

**Table S8. Family-wise correction for multiple comparisons.** Significant results are highlighted in bold.

| **Volume** | | | |
| --- | --- | --- | --- |
| **Tract** | **Estimate** | **Z-value** | **Corrected p-value** |
| Left SLF 1 | .255 | 2.637 | **0.038** |
| Right SLF 1 | 0.039 | 0.659 | 0.999 |
| Left SLF 2 | -0.016 | -0.229 | 1 |
| Right SLF 2 | 0.095 | 1.426 | 0.754 |
| Left SLF 3 | 0.129 | 1.778 | 0.472 |
| Right SLF 3 | 0.038 | 0.560 | 0.999 |
| Left AT | -0.068 | -1.631 | 0.588 |
| Right AT | -0.003 | -0.074 | 1 |
| Left FS | 0.014 | 0.355 | 1 |
| Right FS | 0.023 | 0.605 | 0.999 |
| **Hindrance modulated orientational anisotropy (HMOA)** | | | |
| **Tract** | **Estimate** | **Z-value** | **Corrected p-value** |
| Left SLF 1 | -32.082 | -0.687 | 0.997 |
| Right SLF 1 | -22.708 | -0.485 | 1 |
| Left SLF 2 | -35.674 | -1.178 | 0.894 |
| Right SLF 2 | 10.405 | 0.431 | 1 |
| Left SLF 3 | -8.561 | -0.363 | 1 |
| Right SLF 3 | -8.183 | -0.309 | 1 |
| Left AT | -101.364 | -2.095 | 0.228 |
| Right AT | -30.728 | -0.732 | 0.996 |
| Left FS | -33.092 | -0.692 | 0.997 |
| Right FS | -2.304 | -0.049 | 1 |
| **Lateralization index LI=(R- L)/(R+L)** | | | |
| **Tract** | **Estimate** | **Z-value** | **Corrected p-value** |
| SLF 1 LI | -3.627 | -2.292 | 0.075 |
| SLF 2 LI | 1.359 | 1.215 | 0.708 |
| SLF 3 LI | -2.332 | -1.526 | 0.47 |
| AT LI | 4.029 | 1.792 | 0.286 |
| FS LI | 0.700 | 0.361 | 0.998 |

**Cross-validation**

The 10-fold cross-validation revealed that 70% of the new observations kept the association between left SLF I volume and treatment response, as 55% for the SLF I lateralization index, and 61.6% for the left anterior thalamic HMOA.

**Group comparison - SLF lateralization index and left anterior thalamic HMOA**

Considering the definition of treatment response based on k-mean clustering, non-responders and controls significantly differed in the SLF I lateralization index, (t_(52)_=3.058, p=.004), but not in the left anterior thalamic HMOA (t_(52)_=.267, p=.791). However, responders to treatment and controls did not significantly differ in both these tract metrics (SLF I lateralization index, t_(44)_=.820, p=.417; left anterior thalamic HMOA, t_(44)_=-1.774, p=.083). That is, the anatomy of participants who responded to treatment did not significantly differ from that of controls in these metrics (Fig. 2).

**Lasso regression**

We used lasso regression to identify a profile of associates of treatment response. The identified model achieved 82.5% balanced accuracy and included ten variables. Odd rations are reported in Table S9.

**Table S9. Results of the lasso regression (machine learning).**

| **VARIABLES** | **Odds ratio (OR)** |
| --- | --- |
| Left SLF I volume | 1.0006 |
| Baseline severity of inattentive symptoms (BAARS-INA) | 1.0411 |
| Baseline severity of impulsive/hyperactive symptoms (BAARS-HI) | 1.1935 |
| Medication-naïve status | 1.7772 |
| MPH dose at follow-up | 0.9995 |
| Right handedness (categorical) | 1.0361 |
| Right handedness (dimensional score) | 1.0176 |
| Total IQ | 1.0073 |
| Longest Passivity (under placebo) | 0.9232 |
| Qb impulsivity (under an acute dose of MPH) | 0.8672 |

BAARS-INA= Barkley Adult ADHD Rating Scale-IV -inattentive symptoms; BAARS-IH= Barkley Adult ADHD Rating Scale-IV -impulsive/hyperactive symptoms; IQ= intelligence quotient; Longest Passivity = maximum number of consecutive omission errors;

MPH= methylphenidate; Qb=Qb test.

**REFERENCES**

1. Thiebaut de Schotten M, Dell'Acqua F, Forkel SJ, Simmons A, Vergani F, Murphy DG, et al. A lateralized brain network for visuospatial attention. Nat Neurosci. 2011;14(10):1245-6.

2. Biederman J, Mick E, Surman C, Doyle R, Hammerness P, Harpold T, et al. A randomized, placebo-controlled trial of OROS methylphenidate in adults with attention-deficit/hyperactivity disorder. Biol Psychiatry. 2006;59(9):829-35.

3. Kooij JJS. Adult ADHD : diagnostic assessment and treatment. Third edition. ed. xvii.

4. Muller U, Suckling J, Zelaya F, Honey G, Faessel H, Williams SC, et al. Plasma level-dependent effects of methylphenidate on task-related functional magnetic resonance imaging signal changes. Psychopharmacology (Berl). 2005;180(4):624-33.

5. Smith SM, Jenkinson M, Woolrich MW, Beckmann CF, Behrens TE, Johansen-Berg H, et al. Advances in functional and structural MR image analysis and implementation as FSL. Neuroimage. 2004;23 Suppl 1:S208-19.

6. Andersson JL, Skare S, Ashburner J. How to correct susceptibility distortions in spin-echo echo-planar images: application to diffusion tensor imaging. Neuroimage. 2003;20(2):870-88.

7. Jenkinson M, Smith S. A global optimisation method for robust affine registration of brain images. Med Image Anal. 2001;5(2):143-56.

8. Dell'Acqua F, Scifo P, Rizzo G, Catani M, Simmons A, Scotti G, et al. A modified damped Richardson-Lucy algorithm to reduce isotropic background effects in spherical deconvolution. Neuroimage. 2010;49(2):1446-58.

9. Dell'Acqua F, Simmons A, Williams SC, Catani M. Can spherical deconvolution provide more information than fiber orientations? Hindrance modulated orientational anisotropy, a true-tract specific index to characterize white matter diffusion. Hum Brain Mapp. 2013;34(10):2464-83.

10. Schmahmann JD, Pandya DN, Wang R, Dai G, D'Arceuil HE, de Crespigny AJ, et al. Association fibre pathways of the brain: parallel observations from diffusion spectrum imaging and autoradiography. Brain. 2007;130(Pt 3):630-53.

11. Wedeen VJ, Wang RP, Schmahmann JD, Benner T, Tseng WY, Dai G, et al. Diffusion spectrum magnetic resonance imaging (DSI) tractography of crossing fibers. Neuroimage. 2008;41(4):1267-77.

12. Rojkova K, Volle E, Urbanski M, Humbert F, Dell'Acqua F, Thiebaut de Schotten M. Atlasing the frontal lobe connections and their variability due to age and education: a spherical deconvolution tractography study. Brain Struct Funct. 2016;221(3):1751-66.
